# Construction and validation of a sepsis prediction model using inflammatory and hemodynamic markers in the emergency department

**DOI:** 10.3389/fmed.2026.1837124

**Published:** 2026-07-29

**Authors:** Rong Lu, Jing Zhang, Liang Chen

**Affiliations:** Department of Emergency, Xin Hua Hospital Affiliated to Shanghai Jiao Tong University School of Medicine, Shanghai, China

**Keywords:** early diagnosis, emergency nursing, inflammatory biomarkers, machine learning, prediction model, sepsis

## Abstract

**Objective:**

To develop an early diagnosis prediction model for sepsis in the emergency department by integrating inflammatory, hemodynamic, and nursing assessment indicators.

**Methods:**

A retrospective cohort of 320 patients with suspected infection admitted to our hospital was enrolled. Participants were randomly allocated into a training set and a validation set at a 7:3 ratio. Key variables were selected using least absolute shrinkage and selection operator (LASSO) regression. Subsequently, logistic regression, random forest, and support vector machine models were constructed. Model performance and interpretability were evaluated using the receiver operating characteristic curve, calibration curve, decision curve analysis, and SHapley Additive exPlanations (SHAP) values.

**Results:**

LASSO regression identified three core variables: procalcitonin (PCT), neutrophil-to-lymphocyte ratio (NLR), and Modified Early Warning Score (MEWS). Multivariate analysis revealed that all three were independent risk factors (*p* < 0.05). The random forest model demonstrated an area under the curve (AUC) of 0.737 in the training set and 0.714 in the internal random-split validation set. At the optimal cutoff, the validation performance yielded a sensitivity of 72.7%, specificity of 76.2%, PPV of 61.5%, and NPV of 84.2%. It showed good calibration and provided clinical net benefit. SHAP analysis indicated that MEWS contributed the most to the model’s predictions, followed by NLR and PCT.

**Conclusion:**

The integrated model developed in this study exhibits moderate to satisfactory predictive performance and clinical utility. It may act as a candidate bedside risk-stratification tool pending further external multicenter validation for emergency nursing sepsis screening.

## Introduction

Sepsis is a critical and frequently encountered syndrome in the emergency department (ED), characterized by insidious onset and rapid progression. Early identification and intervention are crucial for improving patient outcomes (1, 2). The ED serves as the frontline for sepsis diagnosis and management. However, the high patient volume and clinical complexity pose a significant challenge: accurately identifying individuals at high risk for sepsis from a large pool of suspected infection cases within a limited time window (3). Current clinical diagnosis relies on physician experience combined with tools like the Sequential Organ Failure Assessment (SOFA), which can be inherently delayed. As the primary assessors upon patient arrival, emergency nurses play a central role in initial screening through early warning scores (e.g., Modified Early Warning Score [MEWS]) and bedside clinical judgment (4, 5). Nevertheless, these nursing assessment tools predominantly rely on vital signs and lack integration with rapidly available biomarkers such as procalcitonin (PCT) and lactate, limiting their predictive performance. In recent years, inflammatory markers and hemodynamic indicators have been demonstrated to be closely associated with the pathophysiology of sepsis, providing an objective basis for early diagnosis (6). Consequently, developing a simple, rapid, and accurate prediction tool suitable for the busy ED environment by effectively integrating nurses’ rapid clinical assessments with these objective laboratory parameters holds significant clinical value. Machine learning methods, capable of handling multidimensional data and uncovering complex relationships, have shown advantages in medical prediction (7, 8). Therefore, this retrospective study aims to identify key predictors and to develop and validate an early diagnostic prediction model for sepsis that incorporates inflammatory markers, hemodynamic parameters, and nursing assessment variables. The goal is to provide an efficient bedside risk-stratification tool for ED nursing and medical teams, thereby facilitating early warning and precise management of sepsis.

## Materials and methods

### Study participants

This retrospective diagnostic study consecutively enrolled adult patients (aged ≥18 years) with clinically suspected infection who presented to our hospital’s ED between January 1, 2023 to June 30, 2024. The study protocol was approved by the hospital’s Ethics Review Committee.

The sample size was calculated based on the empirical rule requiring at least 10 outcome events per estimated predictor variable in predictive model development. With approximately 7 candidate predictors considered and an expected sepsis incidence of 30–40% as the primary outcome, the minimum sample size was estimated using PASS 15.0 software. Setting *α* = 0.05, power at 80%, and accounting for a 15% rate of incomplete data, the estimated minimum sample size was 280. The final enrollment of 320 participants exceeded this pre-calculated minimum sample size to ensure basic statistical power under strict inclusion and exclusion criteria, and 113 sepsis-positive outcome cases (35.3%) were contained in the whole cohort; limited by complete clinical and laboratory data requirements for modeling.

Inclusion criteria: (1) Age ≥18 years; (2) ED visit due to suspected infection; (3) Completion of an ED sepsis screening panel within 2 h of arrival, including inflammatory markers, lactate, and initial hemodynamic assessment; (4) Availability of complete clinical data.

Exclusion criteria: (1) Clear diagnosis of a non-infectious disease; (2) Presentation with established septic shock requiring immediate resuscitation; (3) Direct transfer from other hospital wards or facilities without undergoing initial ED assessment; (4) Missing key laboratory or assessment data; (5) Pregnancy or lactation.

Initially, a total of 1,146 patients with suspected infection were initially screened in our tertiary emergency department. Among these, 320 patients met all eligibility criteria and were finally enrolled, while 826 patients were excluded mainly due to incomplete laboratory data, missing nursing assessment records, age <18 years, established septic shock on admission, transfer from other hospitals without initial ED assessment, non-infectious diseases, or pregnancy/lactation. The relatively modest sample size was attributed to the strict inclusion and exclusion criteria requiring complete data for reliable predictive modeling, which helps ensure data quality but may limit generalizability.

### Data collection

Data were extracted from the hospital’s ED electronic medical record system. Biochemical and inflammatory indicators were tested in the hospital’s accredited clinical laboratory using standard procedures and automatically recorded in the laboratory information system and nursing assessment records, covering the following dimensions: demographics and baseline clinical data: age, sex, number of chronic comorbidities. Inflammatory and infection biomarkers: PCT, C-reactive protein (CRP), neutrophil-to-lymphocyte ratio (NLR), interleukin-6 (IL-6) levels. Hemodynamic and tissue perfusion indicators: lactate level, mean arterial pressure (MAP), shock index (SI), capillary refill time (CRT), central venous oxygen saturation (ScvO₂, if central venous access was established). Organ function and coagulation parameters: acute kidney injury (AKI) stage, platelet count, international normalized ratio (INR), total bilirubin, D-dimer level. Nursing-led clinical assessment and early warning: body temperature, respiratory rate, and consciousness level assessed and documented by the responsible nurse within 1 h of arrival and before laboratory results were available. These were used to calculate MEWS and NEWS scores. The nurse’s bedside comprehensive impression score (0–10 points) and the integrated judgment of “need for urgent intervention” were also recorded.

### Grouping criteria

Initial stratification was based on the ED nursing preliminary risk assessment protocol, and patients were classified into two initial triage categories (distinct from the final sepsis diagnosis): the Nursing High-Risk Group met any of the following criteria—(1) NEWS ≥7, (2) Nurse’s bedside comprehensive impression score ≥7, (3) Initial lactate >2.0 mmol/L, or (4) Presence of definite prolonged capillary refill time (>3 s)—while the Routine Observation Group included patients with suspected infection who did not meet the above criteria. The final diagnostic outcome (sepsis) was independently adjudicated by two senior ED physicians blinded to the initial risk assessment, according to the Sepsis-3 criteria, which defined sepsis as suspected infection combined with an acute increase in the Sequential Organ Failure Assessment (SOFA) score of ≥2 points. In this study, all enrolled patients were assumed to have a baseline SOFA score of 0 due to the absence of documented pre-existing organ dysfunction. SOFA scores were calculated within 6 h of ED arrival using the worst available values for respiratory (PaO₂/FiO_2_ ratio), coagulation (platelet count), liver (total bilirubin), cardiovascular (mean arterial pressure or need for vasopressors), renal (creatinine or urine output), and central nervous system (Glasgow Coma Scale) components, and the assessment was performed by attending physicians using routine ED laboratory and vital sign data. Any disagreement between the two adjudicators was resolved by a third chief physician.

### Statistical analysis

Data were analyzed using SPSS 26.0 and Python 3.9. Normally distributed continuous data are presented as mean ± standard deviation and compared using the independent samples *t*-test. Non-normally distributed data are presented as median (interquartile range) and compared using the Mann–Whitney U test. Categorical data are presented as number (percentage) and compared using the chi-square test. We performed complete-case analysis in this study. No missing data were present for the key variables included in the modeling.

In the training set, univariate analysis was first performed to screen variables associated with sepsis diagnosis (*p* < 0.05). To identify core predictors and avoid overfitting, variables significant in univariate analysis were entered into least absolute shrinkage and selection operator (LASSO) regression. The optimal penalty coefficient (*λ*.min) was determined via 10-fold cross-validation, and variables with non-zero coefficients were retained as core predictors. These LASSO-selected variables were then incorporated into multivariable logistic regression to calculate odds ratios (ORs) and 95% confidence intervals (CIs), confirming independent predictive factors. Multicollinearity was evaluated using variance inflation factor (VIF), and all variables had VIF < 3, indicating no severe multicollinearity.

Based on the core variables, three machine learning models were constructed: logistic regression, random forest, and support vector machine. Hyperparameters were optimized using grid search on the training set. Model performance was evaluated using the receiver operating characteristic (ROC) curve for discrimination. To address the limitation of a single internal random-split validation split, we performed 1,000-fold bootstrapping resampling across the entire dataset. Optimism-corrected AUCs were calculated to assess model stability. The bootstrap-derived 95% CIs and optimism estimates were reported. Calibration curves assessed agreement between predicted and observed probabilities. Decision curve analysis (DCA) evaluated clinical net benefit. Finally, the SHapley Additive exPlanations (SHAP) framework was employed for interpretability analysis of the optimal model, and a nomogram was developed. All tests were two-tailed, with *p* < 0.05 considered statistically significant.

## Results

### Comparison of general characteristics between training and validation sets

Among the 320 included participants, a total of 113 (35.3%) were finally diagnosed with sepsis, with 80 (35.7%) in the training set (*n* = 224) and 33 (34.4%) in the validation set (*n* = 96). No statistically significant differences (*p* > 0.05) were observed between the training set and the validation set regarding demographic characteristics (age, sex), comorbidity profile (number of chronic diseases), inflammatory markers (PCT, CRP, NLR, IL-6), hemodynamic and tissue perfusion indicators (lactate, MAP, SI, CRT, ScvO₂), organ function parameters (AKI stage, platelet count, INR, total bilirubin, D-dimer), or clinical assessment and outcomes (MEWS, NEWS, nurse’s bedside comprehensive impression score, judgment of need for urgent intervention, final sepsis confirmation rate). These findings indicate balanced dataset partitioning and good comparability (Table 1).

**Table 1:** Comparison of general characteristics between training and validation sets.

Variables	Training set (*n* = 224)	Validation set (*n* = 96)	*t*/*χ*^2^	*p*
Age (years)	67.50 ± 16.20	65.80 ± 15.70	0.868	0.386
Sex, *n* (%)			0.039	0.843
Male	128 (57.14)	56 (58.33)		
Female	96 (42.86)	40 (37.67)		
Number of comorbidities	2.20 ± 1.50	2.10 ± 1.40	0.557	0.578
PCT (ng/mL)	5.80 ± 8.20	5.30 ± 7.60	0.511	0.610
CRP ≥ 50 mg/L, *n* (%)			0.111	0.739
Yes	145 (64.73)	64 (66.67)		
No	79 (35.27)	32 (33.33)		
NLR	12.50 ± 8.60	11.90 ± 7.90	0.586	0.559
IL-6 > 7 pg./mL, *n* (%)			0.001	1.000
Yes	98 (43.75)	42 (43.75)		
No	126 (56.25)	54 (56.25)		
Lactate >2.0 mmol/L, *n* (%)			0.319	0.572
Yes	101 (45.09)	40 (41.67)		
No	123 (54.91)	56 (58.33)		
MAP (mmHg)	78.00 ± 15.00	76.00 ± 14.00	1.115	0.266
SI ≥ 0.7, *n* (%)			0.231	0.631
Yes	167 (74.55)	74 (77.08)		
No	57 (25.45)	22 (22.92)		
Capillary refill time >3 s, *n* (%)			0.001	0.981
Yes	110 (49.11)	47 (48.96)		
No	114 (50.89)	49 (51.04)		
ScvO_2_ < 70%, *n* (%)			0.057	0.811
Yes	67 (29.91)	30 (31.25)		
No	157 (70.09)	66 (68.75)		
AKI (KDIGO 1–3), *n* (%)			0.335	0.563
Yes	87 (38.84)	34 (35.42)		
No	137 (61.16)	62 (64.58)		
Platelet count (×10^9^/L)	185.00 ± 102.00	178.00 ± 98.00	0.569	0.570
INR > 1.2, *n* (%)			0.105	0.747
Yes	89 (39.73)	40 (41.67)		
No	135 (60.27)	56 (58.33)		
Total bilirubin >17.1 μmol/L, *n* (%)			0.055	0.814
Yes	73 (32.59)	30 (31.25)		
No	151 (67.41)	66 (68.75)		
D-dimer >0.5 mg/L, *n* (%)			0.059	0.809
Yes	158 (70.54)	69 (71.88)		
No	66 (29.46)	27 (28.12)		
MEWS	3.50 ± 2.10	3.40 ± 2.00	0.396	0.692
Temperature abnormality (<36 °C or >38.3°C), *n* (%)			0.202	0.653
Yes	134 (59.82)	60 (62.50)		
No	90 (40.18)	36 (37.50)		
Respiratory rate (breaths/min)	24.00 ± 6.00	25.00 ± 7.00	1.298	0.195
Altered mental status (GCS < 15), *n* (%)			0.220	0.639
Yes	71 (31.70)	33 (34.38)		
No	153 (68.30)	63 (65.62)		
NEWS ≥7, *n* (%)			1.633	0.201
Yes	152 (67.86)	72 (75.00)		
No	72 (32.14)	24 (25.00)		
Nurse bedside impression score (0–10)	6.20 ± 2.50	6.10 ± 2.40	0.332	0.740
Judgment requiring urgent intervention, *n* (%)			0.040	0.842
Yes	135 (60.27)	59 (61.46)		
No	89 (39.73)	37 (38.54)		

PCT, procalcitonin; CRP, C-reactive protein; IL-6, interleukin-6; ScvO_2_, central venous oxygen saturation; AKI, acute kidney injury; INR, international normalized ratio; MEWS, Modified Early Warning Score; NEWS, National Early Warning Score.

### Characteristic comparison between sepsis group and non-sepsis group

In the training set, patients were divided into the sepsis group (*n* = 80) and the non-sepsis group (*n* = 144). Comparative analysis showed that the sepsis group exhibited significantly higher levels of PCT, NLR, and MEWS, while the proportions of patients with lactate >2.0 mmol/L and capillary refill time >3 s were significantly higher compared to the non-sepsis group (*p* < 0.05). (Supplementary Table 1).

### Variable screening via LASSO regression

**Figure 1:**
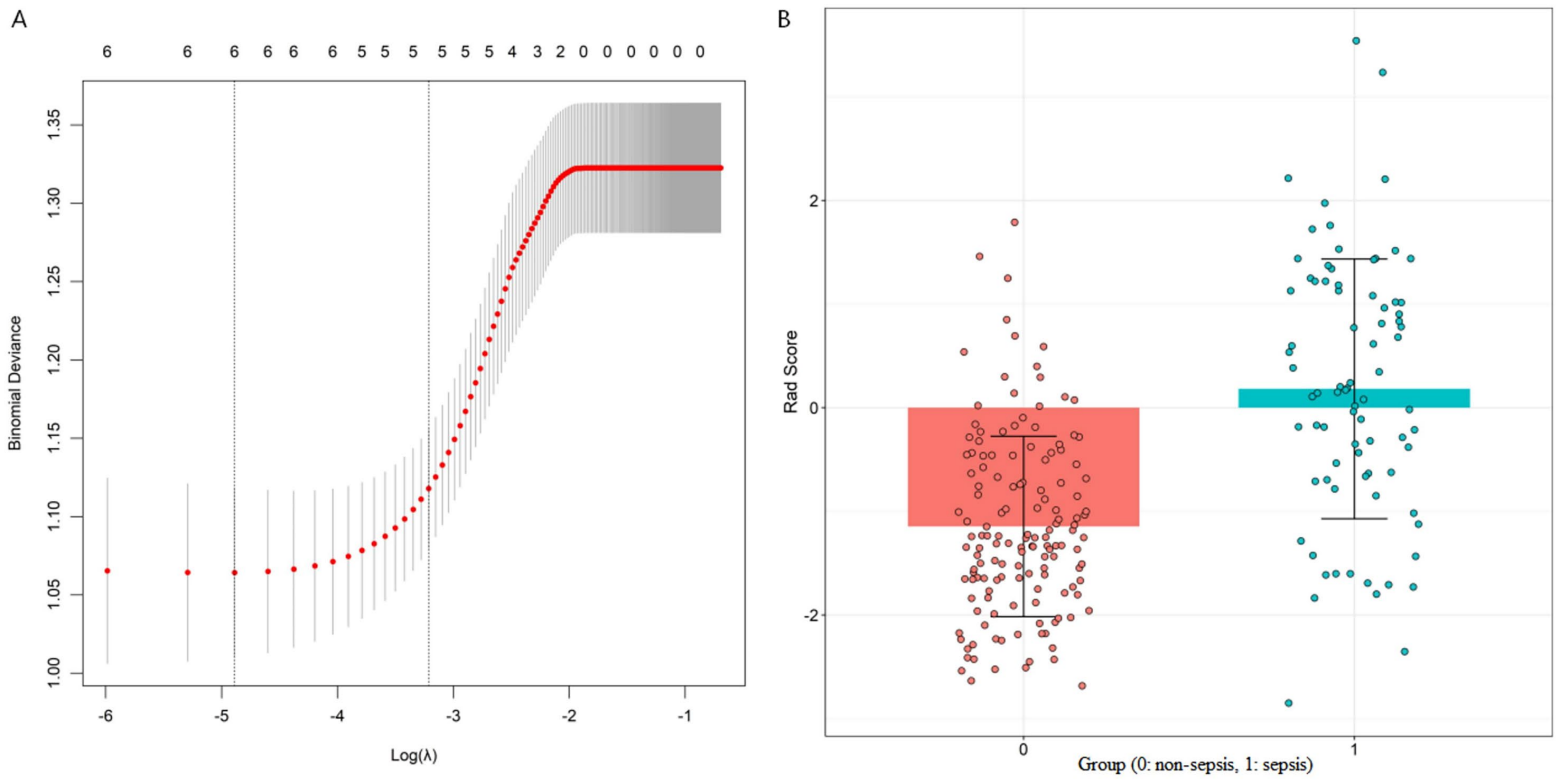
10-fold cross-validation plot for optimal *λ* selection **(A)** and dot plot comparing Rad Scores between the sepsis and non-sepsis groups **(B)**. **(A)** The *x*-axis represents Log(*λ*) (logarithm of the penalty parameter), and the *y*-axis represents binomial deviance. The optimal penalty parameter *λ*.min was determined by minimizing the binomial deviance via 10-fold cross-validation. **(B)** The *x*-axis represents the group (0: non-sepsis, 1: sepsis), and the *y*-axis represents the Rad Score.

To identify core predictive variables and avoid overfitting, variables demonstrating statistical significance in the univariate analysis were subjected to LASSO regression analysis. The optimal penalty coefficient (*λ*) was determined using 10-fold cross-validation. The LASSO coefficient path plot illustrates the gradual shrinkage of variable coefficients as Log(*λ*) increases; model performance was optimized at a specific Log(λ) value (Figures 1A,B). Consequently, three core predictive variables were selected for model construction: PCT, NLR, and the MEWS.

### Multivariate logistic regression analysis

The three variables selected by LASSO regression were incorporated into a multivariate logistic regression model (Supplementary Table 2). The results indicated that PCT (OR = 1.153, 95% CI: 1.060–1.254), NLR (OR = 1.084, 95% CI: 1.039–1.132), and the MEWS (OR = 1.191, 95% CI: 1.020–1.391) were independent risk factors for sepsis diagnosis (Table 2).

**Table 2:** Multivariate logistic regression analysis for early sepsis diagnosis prediction in the emergency department.

Variable	*β*	SE	Wald	*p*	OR	95%CI
PCT	0.142	0.043	10.891	0.001	1.153	1.060–1.254
NLR	0.081	0.022	13.556	<0.001	1.084	1.039–1.132
MEWS	0.175	0.079	4.902	0.027	1.191	1.020–1.391

PCT, procalcitonin; NLR, neutrophil-to-lymphocyte ratio; MEWS, Modified Early Warning Score.

### Performance evaluation of machine learning models

**Figure 2:**
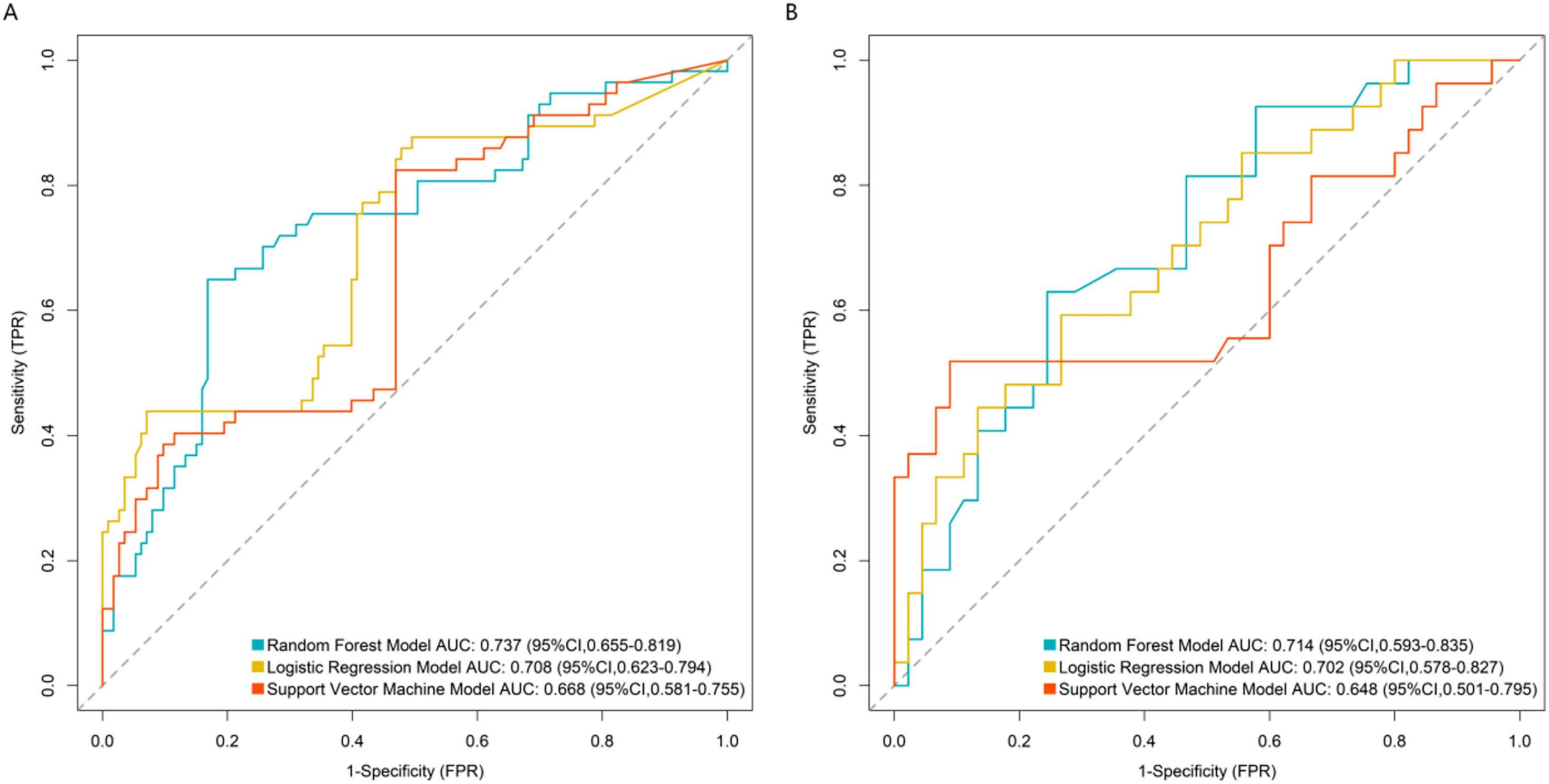
Receiver operating characteristic curve analysis of the prediction model in the training **(A)** and validation **(B)** sets. The *x*-axis represents 1-specificity (FPR, false positive rate), and the *y*-axis represents sensitivity (TPR, true positive rate).

ROC analysis showed random forest obtained the highest AUC of 0.737 (95% CI: 0.655–0.819) in the training cohort, followed by logistic regression (0.708, 95% CI: 0.623–0.794) and support vector machine (0.668, 95% CI: 0.581–0.755). In the validation set, the corresponding AUCs were 0.714 (95% CI: 0.593–0.835) for random forest, 0.702 (95% CI: 0.578–0.827) for logistic regression and 0.648 (95% CI: 0.501–0.795) for support vector machine (Figures 2A,B). After bootstrapping optimism correction, the bias-corrected AUCs were adjusted correspondingly to 0.722 (95% CI: 0.641–0.803) for random forest, 0.692 (95% CI: 0.607–0.777) for logistic regression, and 0.656 (95% CI: 0.567–0.745) for support vector machine, with consistent optimism estimates of 0.015, 0.016, and 0.012. These corrected AUCs closely approximated the validation set AUCs, with differences ≤0.023 for all models, indicating that the models did not suffer from substantial overfitting and that the initial performance estimates were not overly optimistic. Using the Youden index to calculate the optimal threshold based on the training set, the performance metrics were then evaluated on the validation set. The random forest model achieved a sensitivity of 72.7%, specificity of 76.2%, positive predictive value (PPV) of 61.5%, and negative predictive value (NPV) of 84.2%; the logistic regression model achieved a sensitivity of 65.6%, specificity of 73.0%, PPV of 56.8%, and NPV of 79.3%; and the support vector machine achieved a sensitivity of 60.6%, specificity of 68.3%, PPV of 50.0%, and NPV of 76.8%. The detailed confusion matrices for these calculations are provided in Supplementary Table 3.

**Figure 3:**
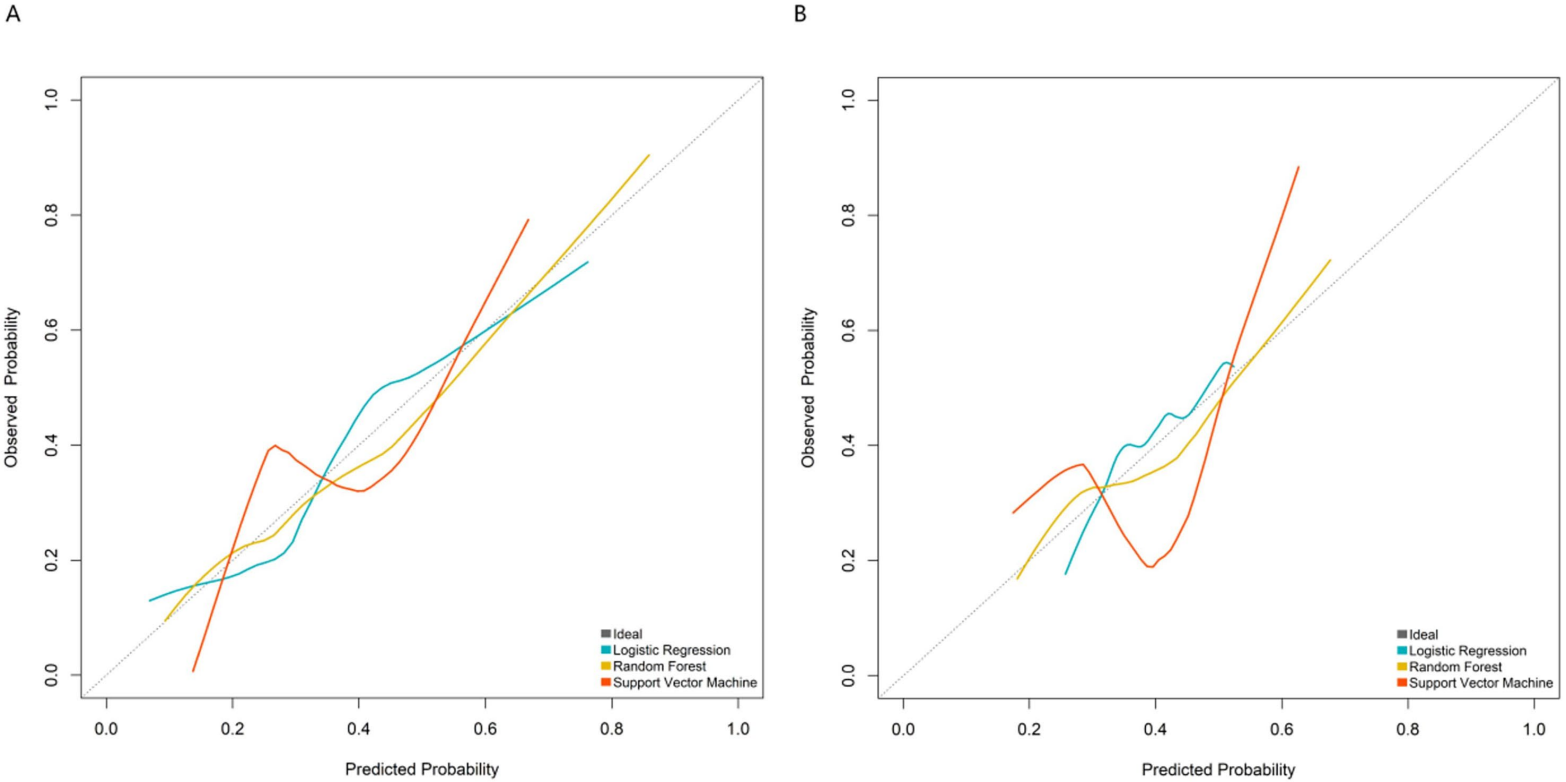
Calibration curve analysis of the prediction models in the training set **(A)** and validation set **(B)**. The *x*-axis represents the predicted probability of sepsis; the *y*-axis represents the observed probability of sepsis. The diagonal line indicates perfect calibration.

Calibration curves (Figures 3A,B) presented moderately favorable consistency between predicted and actual risks for logistic regression and random forest, whereas support vector machine only achieved moderate calibration in the validation group. The random forest model yielded a Brier score of 0.193 in the training set and 0.215 in the validation set, with calibration slopes of 0.921 and 0.897, and calibration intercepts of −0.12 and −0.18, respectively. At the optimal cutoff value (0.385, determined by the Youden index in the training set), its positive likelihood ratio (LR+) was 3.06 and negative likelihood ratio (LR−) was 0.36 in the validation set. The detailed confusion matrices, cutoff values, and calibration parameters for all three models are summarized in Supplementary Table 3. The logistic regression model had Brier score 0.211 (training set)/0.234(validation set), calibration slope 0.903/0.872, LR+=2.46, LR−=0.39; support vector machine had Brier score 0.239(training set)/0.258(validation set), calibration slope 0.859/0.821, LR+=1.96, LR−=0.48.

**Figure 4:**
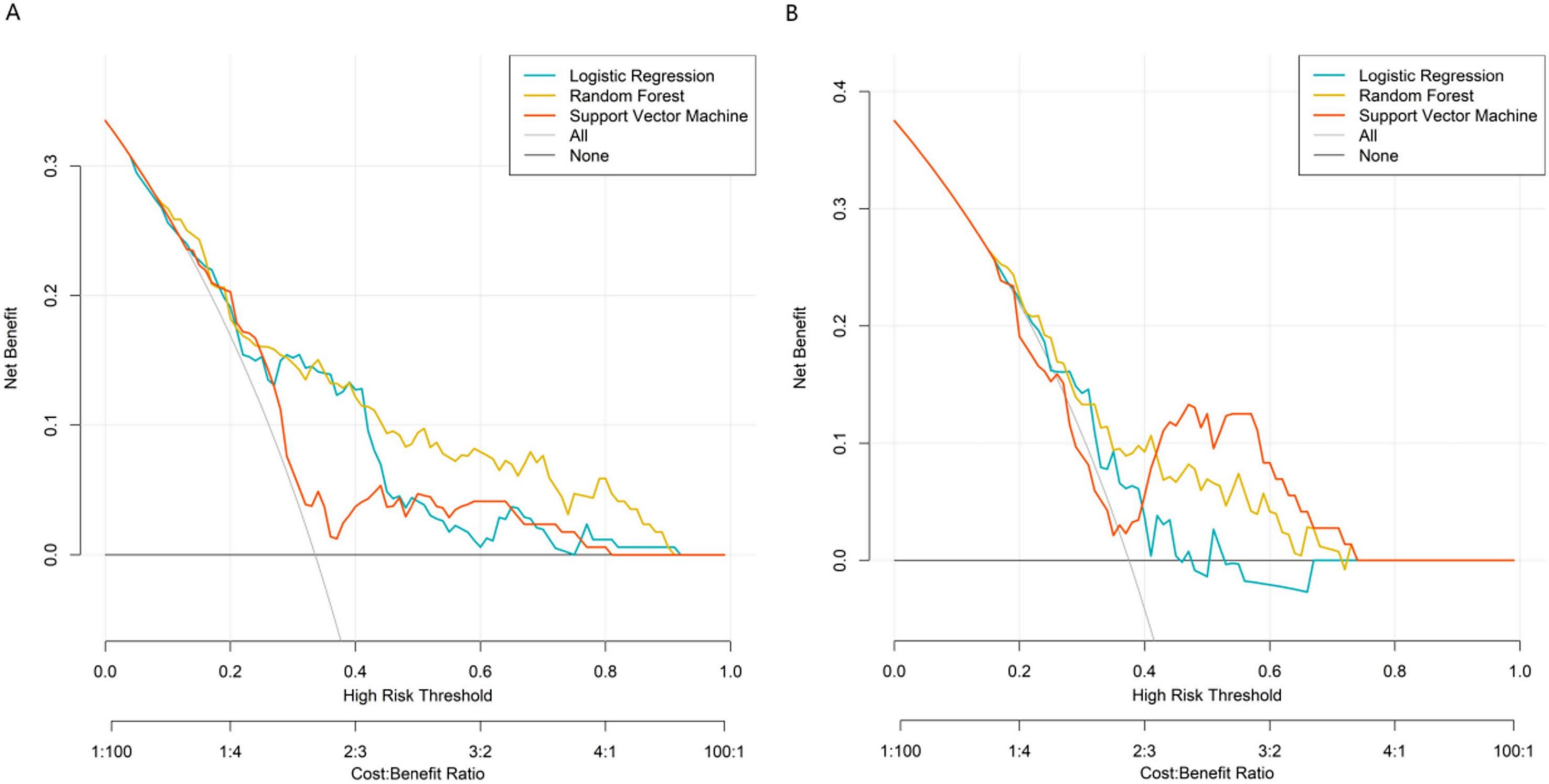
Clinical decision curve analysis of the prediction model in the training **(A)** and validation **(B)** sets. The *x*-axis represents the threshold probability of sepsis; the *y*-axis represents the net benefit. The “All” line indicates the assumption that all patients are treated; The “None” line indicates that no patients are treated.

DCA curves for the training set (Figure 4A) showed that logistic regression and random forest provided positive net benefit across a broad threshold range (approximately 0.05–0.50), with random forest exhibiting a slight advantage between 0.15 and 0.35, while support vector machine yielded net benefit primarily at thresholds above 0.20. In the validation set (Figure 4B), logistic regression and random forest demonstrated comparable positive net benefit within the narrower range of 0.10–0.22, with both converging to near zero beyond 0.22. In contrast, the support vector machine model showed net benefit near or below zero at thresholds below 0.16, but progressively increased from 0.16 to 0.66, and achieved the highest net benefit among all models at thresholds above 0.30.

### Interpretability assessment of model predictions

**Figure 5:**
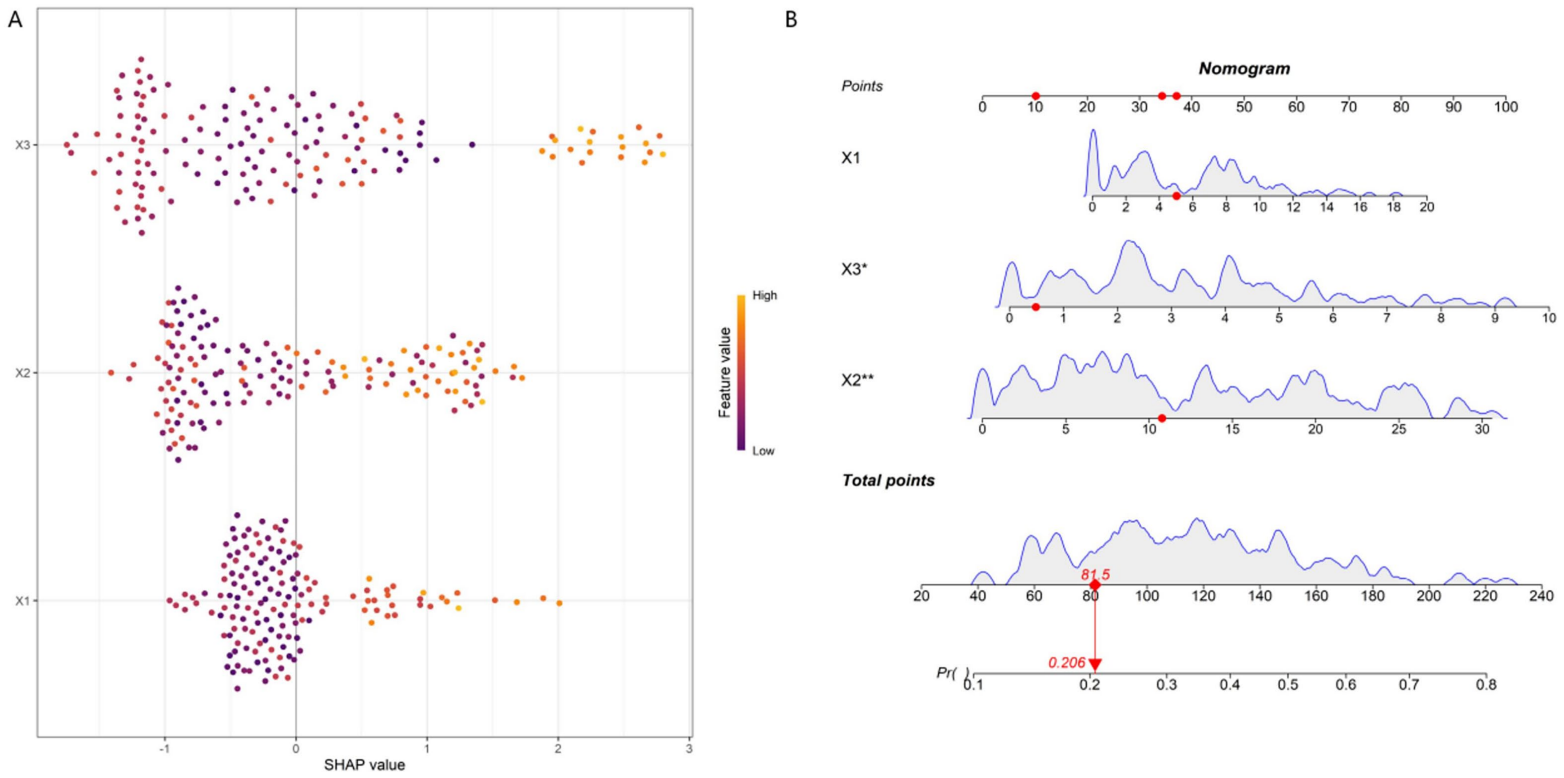
SHAP feature importance plot **(A)** and nomogram **(B)**. **(A)** SHAP values quantify the contribution of each variable to model prediction. MEWS shows the highest impact, followed by NLR and PCT; **(B)** The nomogram was constructed based on logistic regression for clinical bedside use. Points are assigned for each variable, and the total points correspond to the individual probability of sepsis.

SHAP values were employed to assess the interpretability of the random forest model, which demonstrated superior performance. The SHAP feature importance plot (Figure 5A) identified the following order of variable contribution to predictions: MEWS (X3), NLR (X2), PCT (X1). Furthermore, a nomogram was constructed based on the logistic regression model for clinical usability, while the random forest model showed the best predictive performance (Figure 5B) to visually represent the contribution of each variable to an individual’s sepsis risk and the corresponding predicted probability based on the total points, facilitating rapid clinical assessment.

## Discussion

Early recognition of sepsis is crucial for reducing mortality in the ED, yet diagnosis is often delayed due to its non-specific presentation (9, 10). Focusing on patients with suspected infection in the ED, this study innovatively integrated rapid clinical assessment parameters (MEWS and capillary refill time) collected by nursing staff with readily available objective inflammatory and hemodynamic markers. Consequently, we successfully developed and validated three machine learning prediction models: logistic regression, random forest, and support vector machine (11). The main findings are as follows. First, the three-variable combination selected by LASSO regression demonstrated favorable predictive performance, with PCT, NLR, and MEWS identified as independent risk factors for sepsis diagnosis. Second, the random forest model exhibited relatively stable and moderate-to-satisfactory discriminative ability in both the training and internal random-split validation sets (AUCs of 0.737 and 0.714 respectively). Third, DCA confirmed the clinical utility of the model across a wide range of risk thresholds. Fourth, SHAP analysis enhanced the model’s interpretability by clarifying the contribution of each feature, revealing that MEWS had the highest impact on model predictions, followed by NLR and PCT. Based on these insights, a clinically practical nomogram was subsequently constructed (12, 13). It is worth noting that the SHAP ranking in the non-linear random forest model differs from the OR ranking observed in logistic regression; this discrepancy is expected, as SHAP captures complex interactions and non-linear associations, whereas logistic regression assumes a linear, additive relationship.

The MEWS, a concise bedside tool focusing primarily on circulatory and consciousness status, demonstrated the highest predictive contribution (OR = 1.191, 95% CI: 1.020–1.391) per SHAP analysis, underscoring that early hemodynamic instability and altered mental status serve as critical warning signs for sepsis. The NLR ranked second in predictive importance (OR = 1.084, 95% CI: 1.039–1.132). The predictive value of PCT, a classic marker of infection and inflammation, was reaffirmed in this study (OR = 1.153, 95% CI: 1.060–1.254), with its contribution ranking third in the SHAP analysis (14). The core predictors identified in this study reflect early pathophysiological alterations in sepsis through different pathways. Elevated NLR reflects not only neutrophil activation but also suggests lymphocyte exhaustion, which is associated with immune dysregulation in sepsis.

Our study confirmed NLR as an independent risk factor (15, 16). Early hemodynamic instability and altered mental status, captured by MEWS, serve as critical warning signs for sepsis (17). Regarding model development and methodology, this study adhered to standard procedures for prediction model research. The use of LASSO regression for feature selection effectively mitigated the risk of overfitting with high-dimensional data, yielding a parsimonious and clinically feasible variable set (18, 19). By comparing algorithms of varying complexity, we found that the random forest model demonstrated good generalizability during internal validation. Decision curve analysis validated the model’s application value from a perspective of clinical net benefit, indicating that using this model to guide clinical decisions (e.g., intensified monitoring or early initiation of bundle therapy) yields greater net benefit compared to strategies of “treating all as high-risk” or “treating all as low-risk” (20).

The implementation of SHAP analysis demystified the “black-box” model, and the creation of a nomogram significantly enhanced the model’s practicality and acceptability in ED nursing practice. Nurses can now rapidly calculate a total score and estimate the risk probability based on the patient’s individual indicators. The innovation of this study lies in its close alignment with the ED nursing workflow, effectively integrating nurse-led rapid, dynamic clinical assessments with objective laboratory markers. This approach transcends traditional models that rely solely on complex scoring systems or single biomarkers. Instead, it develops an integrated prediction tool that better fits the practical realities of the ED and possesses potential for rapid deployment. It emphasizes the frontline value of nursing assessment in early recognition and provides a paradigm for quantifying nursing observation data and incorporating it into predictive algorithms. Nevertheless, this study has several limitations. First, the sample size is modest for machine learning-based prediction modeling, despite meeting the minimum events-per-variable criterion. The validation set is relatively small, which may lead to wider confidence intervals and less precise performance estimates. Therefore, external validation in larger, prospective, multicenter cohorts is essential before clinical deployment. Second, it is a single-center retrospective study; the population characteristics may be influenced by regional factors, further limiting generalizability. Third, the study included variables like central venous oxygen saturation, which are not routinely measured for all ED patients. Future work could explore a simplified version based entirely on data universally available early after admission. Fourth, the model provides a static prediction and does not incorporate dynamic parameters following the initiation of treatment. Future research could explore dynamic prediction models that integrate temporal data and further investigate the impact of this predictive model on clinical outcomes, such as 28-day mortality and ICU transfer rates. In summary, this study developed and preliminarily validated an early diagnosis prediction model for sepsis in the ED by integrating inflammatory markers, hemodynamic indicators, and nursing clinical assessment parameters. The model demonstrates moderate-to-satisfactory predictive performance, calibration, and clinical utility. It not only provides a quantitative tool for the early identification of high-risk sepsis patients in the ED but also highlights the value of systematically integrating nursing assessment data with objective laboratory indicators for early warning of acute critical illnesses. This work contributes to advancing ED sepsis management toward greater efficiency.

## Data Availability

The original contributions presented in the study are included in the article/Supplementary material, further inquiries can be directed to the corresponding author/s.
