# Supplementary material for: Construction and validation of a sepsis prediction model using inflammatory and hemodynamic markers in the emergency department
Source: Front Med (Lausanne). 2026 Jul 29;13:1837124. doi: 10.3389/fmed.2026.1837124 (PMC13461702; doi:10.3389/fmed.2026.1837124)
Supplement: Supplementary file 1 [file Supplementary_file_1.docx]

**Supplemental Table 1.** Comparison of Patient Characteristics in the Training Set Stratified by Nursing Assessment and Objective Indicators

| Variables | Sepsis group (n=80) | Non-sepsis group (n=144) | *t/χ²* | *P* |
| --- | --- | --- | --- | --- |
| Age (years) | 69.20±15.60 | 66.80±16.30 | 1.072 | 0.285 |
| Sex, n (%) |  |  | 0.041 | 0.841 |
| Male | 45 (56.25) | 83 (57.64) |  |  |
| Female | 35 (43.75) | 61 (42.36) |  |  |
| Number of comorbidities | 2.25±1.55 | 2.18±1.48 | 0.334 | 0.739 |
| PCT (ng/mL) | 6.50±5.25 | 4.85±3.45 | 2.831 | **0.005** |
| CRP ≥50 mg/L, n (%) |  |  | 3.289 | 0.070 |
| Yes | 58 (72.50) | 87 (60.42) |  |  |
| No | 22 (27.50) | 57 (39.58) |  |  |
| NLR | 15.20±9.10 | 10.80±7.35 | 3.936 | **0.001** |
| IL‑6 >7 pg/mL, n (%) |  |  | 0.711 | 0.399 |
| Yes | 38 (47.50) | 60 (41.67) |  |  |
| No | 42 (52.50) | 84 (58.33) |  |  |
| Lactate >2.0 mmol/L, n (%) |  |  | 9.627 | **0.002** |
| Yes | 25 (31.25) | 76 (52.78) |  |  |
| No | 55 (68.75) | 68 (47.22) |  |  |
| MAP (mmHg) | 77.20±14.50 | 78.65±15.25 | 0.694 | 0.489 |
| SI ≥0.7, n (%) |  |  | 0.189 | 0.664 |
| Yes | 61 (76.25) | 106 (73.61) |  |  |
| No | 19 (23.75) | 38 (26.39) |  |  |
| Capillary refill time >3 s, n (%) |  |  | 6.709 | **0.010** |
| Yes | 30 (37.50) | 80 (55.56) |  |  |
| No | 50 (62.50) | 64 (44.44) |  |  |
| ScvO₂ <70%, n (%) |  |  | 0.001 | 0.982 |
| Yes | 24 (30.00) | 43 (29.86) |  |  |
| No | 56 (70.00) | 101 (70.14) |  |  |
| AKI (KDIGO 1–3), n (%) |  |  | 0.702 | 0.402 |
| Yes | 34 (42.50) | 53 (36.81) |  |  |
| No | 46 (57.50) | 91 (63.19) |  |  |
| Platelet count (×10⁹/L) * | 180.25±96.50 | 186.75±104.80 | 0.457 | 0.648 |
| INR >1.2, n (%) |  |  | 0.120 | 0.729 |
| Yes | 33 (41.25) | 56 (38.89) |  |  |
| No | 47 (58.75) | 88 (61.11) |  |  |
| Total bilirubin >17.1 μmol/L, n (%) |  |  | 0.329 | 0.566 |
| Yes | 28 (35.00) | 45 (31.25) |  |  |
| No | 52 (65.00) | 99 (68.75) |  |  |
| D‑dimer >0.5 mg/L, n (%) |  |  | 0.231 | 0.631 |
| Yes | 58 (72.50) | 100 (69.44) |  |  |
| No | 22 (27.50) | 44 (30.56) |  |  |
| MEWS | 3.75±2.80 | 2.85±1.60 | 3.063 | **0.003** |
| Temperature abnormality (<36°C or >38.3°C), n (%) |  |  | 0.106 | 0.745 |
| Yes | 49 (61.25) | 85 (59.03) |  |  |
| No | 31 (38.75) | 59 (40.97) |  |  |
| Respiratory rate (breaths/min) | 24.60±6.15 | 23.80±5.95 | 0.953 | 0.342 |
| Altered mental status (GCS <15), n (%) |  |  | 0.242 | 0.623 |
| Yes | 27 (33.75) | 44 (30.56) |  |  |
| No | 53 (66.25) | 100 (69.44) |  |  |
| NEWS ≥7, n (%) |  |  | 5.305 | **0.021** |
| Yes | 62 (77.50) | 90 (62.50) |  |  |
| No | 18 (22.50) | 54 (37.50) |  |  |
| Nurse bedside impression score (0–10) | 6.40±2.35 | 6.05±2.55 | 1.012 | 0.313 |
| Judgment requiring urgent intervention, n (%) |  |  | 1.164 | 0.281 |
| Yes | 52 (65.00) | 83 (57.64) |  |  |
| No | 28 (35.00) | 61 (42.36) |  |  |

Abbreviation: PCT, procalcitonin; CRP, C-reactive protein; IL-6, interleukin-6; ScvO₂, central venous oxygen saturation; AKI, acute kidney injury; INR, international normalized ratio; MEWS, Modified Early Warning Score.

**Supplemental Table 2.** Variable Assignment Table.

| Variable | Meaning | Assignment |
| --- | --- | --- |
| X1 | PCT | Continuous variable |
| X2 | NLR | Continuous variable |
| X3 | Lactate | 1=Yes,2=No |
| X4 | Capillary Refill Time >3 seconds | 1=Yes,2=No |
| X5 | MEWS | Continuous variable |
| X6 | NEWS ≥7 | 1=Yes,2=No |
| Y | Finally diagnosed as sepsis | 1=Yes,2=No |

Abbreviation: PCT, procalcitonin; NLR, neutrophil-to-lymphocyte ratio; MEWS, Modified Early Warning Score; NEWS, National Early Warning Score.

**Supplementary Table 3.** Confusion Matrices and Performance Metrics of the Three Models in the Validation Set.

| Model | Confusion Matrix | Sensitivity | Specificity | PPV | NPV | Youden's Index | Optimal Cutoff |
| --- | --- | --- | --- | --- | --- | --- | --- |
| Random Forest | TP=24, FN=9  FP=15, TN=48 | 72.7%  (24/33) | 76.2%  (48/63) | 61.5%  (24/39) | 84.2%  (48/57) | 0.489 | 0.385 |
| Logistic Regression | TP=22, FN=11  FP=17, TN=46 | 66.7%  (22/33) | 73.0%  (46/63) | 56.4%  (22/39) | 80.7%  (46/57) | 0.397 | 0.372 |
| Support Vector Machine | TP=20, FN=13  FP=20, TN=43 | 60.6%  (20/33) | 68.3%  (43/63) | 50.0%  (20/40) | 76.8%  (43/56) | 0.289 | 0.341 |

Abbreviations: TP, true positive; FN, false negative; FP, false positive; TN, true negative; PPV, positive predictive value; NPV, negative predictive value. Optimal cutoff values were determined by the Youden index in the training set. The validation set contained 33 sepsis cases (34.4%) and 63 non-sepsis cases (65.6%). All performance metrics are reported on the validation set.
